# Comprehensive integrative analysis of circadian rhythms in human saliva

**DOI:** 10.1038/s44323-025-00035-3

**Published:** 2025-05-10

**Authors:** Nina Nelson, Deeksha Malhan, Janina Hesse, Ouda Aboumanify, Müge Yalçin, Georg Lüers, Angela Relógio

**Affiliations:** 1 Institute for Systems Medicine and Faculty of Human Medicine, MSH Medical School Hamburg, Hamburg, Germany; 2 Leibniz Institute for Resilience Research (LIR), Mainz, Germany and Johannes Gutenberg University Medical Center, Mainz, Germany; 3 Molecular Cancer Research Center (MKFZ), Medical Department of Haematology, Oncology, and Tumor Immunology, Charité—Universitätsmedizin Berlin, Corporate Member of Freie Universität Berlin Humboldt—Universität zu Berlin, and Berlin Institute of Health, Berlin, Germany; 4 Institute for Theoretical Biology (ITB), Charité—Universitätsmedizin Berlin, Corporate Member of Freie Universität Berlin, Humboldt—Universität zu Berlin, and Berlin Institute of Health, Berlin, Germany; 5 Faculty of Human Medicine, MSH Medical School Hamburg, Hamburg, Germany

**Keywords:** Biological techniques, Cell biology

## Abstract

The circadian clock orchestrates vital physiological functions, with its dysregulation implicated in various pathologies. Assessing human clock status via the measurement of circadian rhythms is crucial for health management and disease treatment. Saliva provides a non-invasive means for such analysis. In this study, we examined circadian rhythms and related parameters in 21 healthy individuals (*n* = 4 – 19 per experiment), assessing different data types, including saliva gene expression with our TimeTeller^®^ methodology, hormone levels, cell composition, and self-assessment tests for chronotype evaluation. While substantial individual variability of the circadian profiles was observed, we found significant correlations between the acrophases of *ARNTL1* gene expression and of cortisol, and both acrophases correlated with the bedtime of individuals on the sampling day. Our findings validate the robustness and reliability of our method for determining peripheral clock circadian rhythms in humans, offering potential for clinical applications in diverse cohorts.

## Introduction

The circadian clock generates ~24 h (hours) oscillations and controls a myriad of physiological and cellular processes in mammals^1^. The central clock in the suprachiasmatic nucleus (SCN) can be directly entrained by light, the strongest zeitgeber (German for “time giver”)^2^. In addition to the central clock in the SCN, peripheral clocks are present in nearly all body cells, susceptible to entrainment by non-photic zeitgebers like food intake and exercise, with tissue-specific effects. The molecular circadian clock machinery consists of translational and transcriptional feedback loops involving a set of core-clock genes and their protein products^3^.

Maintaining a functional circadian rhythm is crucial for humans and other organisms. Mounting evidence links circadian disruption to various diseases, encompassing cancer, pathologies of the cardiovascular system, immune-related disorders, metabolic diseases, neurodegenerative conditions, and mental health disorders^1,4–8^. Characterizing the individual circadian profile is thus crucial for: (1) customizing routines to maintain health and (2) identifying circadian deviations early to prevent disease onset^9^. In addition, a treatment regime optimized to the patient’s circadian profile (chronotherapy) may enhance treatment response and decrease adverse effects^10^. The circadian profile may be influenced by behavior (e.g., alterations in sleep-wake cycles), as well as biological factors like age, sex, and health status^2^.

The chronotype reflects preferences for a later or earlier sleep- and wake-up time and activity peak. It is, therefore, also seen as an estimate of the circadian phase. The chronotype is typically determined by questionnaires, which classify individuals as morning, intermediate, or evening chronotypes depending on score cut-offs^11^. The Morningness-Eveningness-Questionnaire (MEQ) correlates well with circadian markers like Core Body Temperature (CBT) and melatonin onset^12,13^. Originally validated in 18-32-year-old students, Taillard et al. recommended adjusting cut-offs for older populations^14^. While useful for estimating chronotype and sleep preferences, MEQ lacks complexity^15–17^, omitting fluctuations in core-clock genes, hormones, and physiological markers crucial for informed health decisions^11,18^. Various markers are used to assess circadian rhythms. Dim Light Melatonin Onset (DLMO) is the gold standard for phase determination, while cortisol’s reliability is debated^2,19^. Molecular markers, like β-Arrestin (*ARRB1*), show promise. For instance, *ARRB1* may better indicate jetlag shifts than DLMO^20^. Physiological measures such as CBT or sleep-activity assessments are often obscured by daily routine and behavioral variations^2^.

Developing non-invasive, robust, and easy-to-use methods for measuring the full profile of the individual circadian rhythm is fundamental both for research in the context of human chronobiology as well as regarding practical clinical applications. Biological materials to measure circadian rhythms in humans include blood, urine, saliva, breath, and hair follicles^2,21,22^. Saliva represents an ideal starting material as it can be obtained non-invasively in an at-home setting^23^. Saliva is produced by the salivary glands and is important for the homeostasis of the oral cavity. It contains electrolytes, enzymes, hormones, messenger substances, cells, and microorganisms^23^. The composition of saliva shows significant interindividual and temporal variances^24,25^. Saliva-omics includes the genome, transcriptome, proteome, metabolome, microbiome, and exosomes^23^ and holds promise in clinical settings, with saliva harboring potential biomarkers for cancer, inflammatory, and neurological diseases^23^. Collection methods, mainly unstimulated and stimulated whole saliva, may impact proteomic results, but no significant influence was shown for the composition of nucleic acids (DNA and RNA)^23^.

**Fig. 1:**
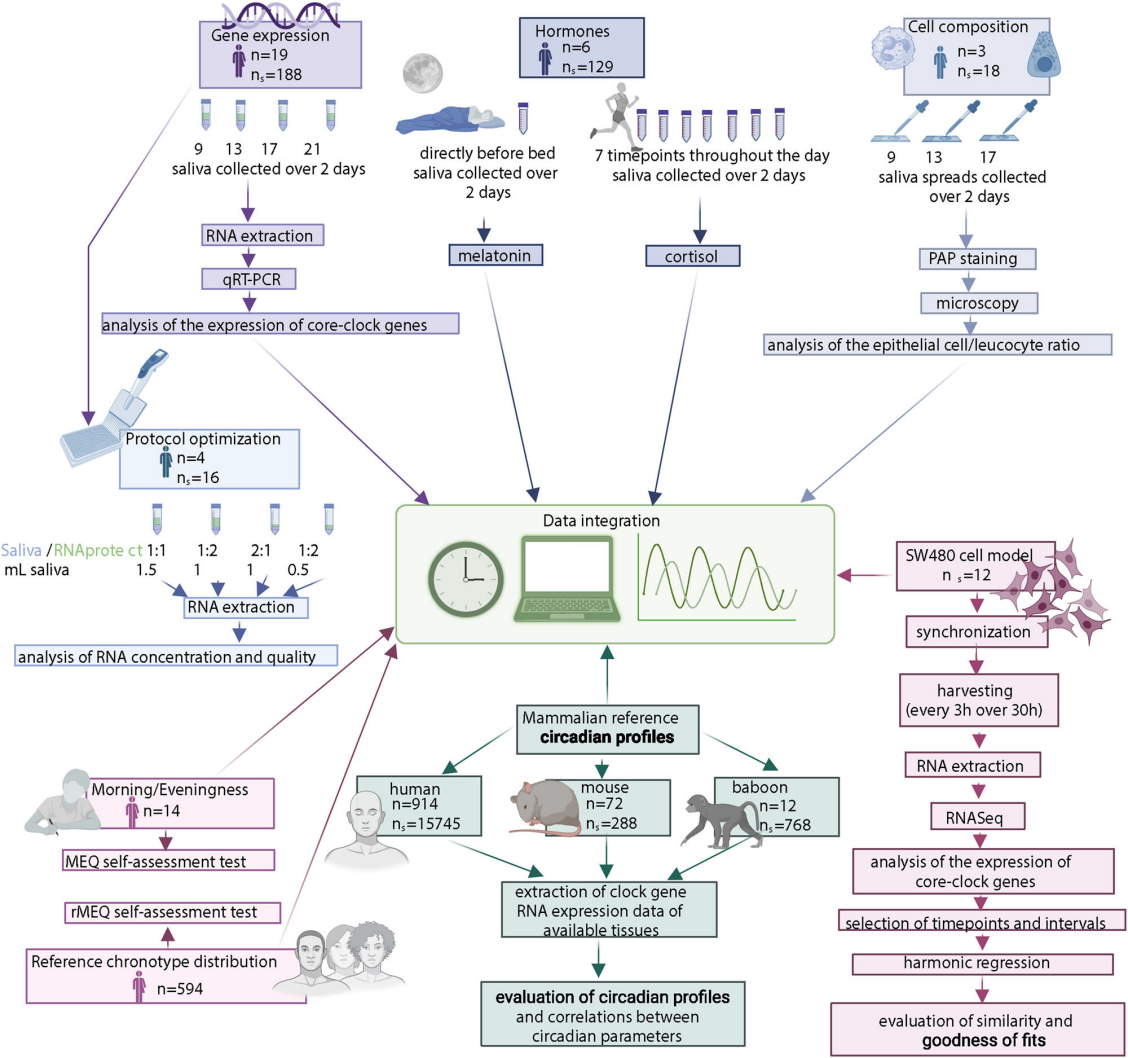
Overview of the study structure. The study consists of multiple methods to assess the circadian rhythm. Experimental data from the study cohort (gene expression, hormones, cell composition), as well as experimental data from cells (SW480 cell model), and publicly available datasets (mammalian reference circadian profiles) were used. *n* = number of participants or animals, *n*_s _= number of samples collected. Created in BioRender. Nelson, N. (2025) https://BioRender.com/e70p177.

Here, we describe a protocol for characterizing the molecular profile of the circadian rhythm in saliva using RNA levels of core-clock genes, hormonal data (cortisol/melatonin), and data on cell composition of collected samples (epithelial cells/leukocytes). We aimed to do a comprehensive assessment of the robustness and comparability of our primary method, gene expression analysis of core clock genes in saliva, while elucidating the cellular landscape from which the saliva RNA, which is being quantified, originates. Whereas we and others have previously assessed time-courses of core-clock gene expression at the RNA level^26–30^, in this study, we integrated a diverse set of data (Fig. 1). To our knowledge, RNA levels of *PER* genes were previously reported to be correlated to cortisol^29^ and RNA levels of *PER1,2* and *NR1D1* to melatonin^28^, however in both studies clock genes and hormones were measured in different biological materials as opposed to our study, which aims to compare them in the same biological material i.e., saliva. In addition, neither hormones nor gene expression of core clock genes have been correlated to variations in cell content. Also, cell content in saliva was previously analyzed for one timepoint^31,32^, but not for a time series as here. By integrating multiple levels of data from a time-course analysis of saliva samples, we demonstrate that our method is robust, user-friendly, and allows for the quantification of circadian rhythms in human saliva samples. This approach offers promising potential for medical applications, ranging from monitoring of circadian rhythms and the detection of related- alterations, to the personalization of medical interventions to the circadian profile of a patient.

## Results

### Study design and main goals

Data were collected from 2018 to 2023 from 21 healthy participants (52.4% female) with an average age of 31 years (25–43 years). The individual experiments consisted of 4 to 19 participants, depending on the experiment, as described below. Saliva was collected at 3–4 time points/day over 2 consecutive days for RNA extraction and core clock gene expression analysis using TimeTeller^®^ kits. Five participants provided saliva samples at multiple time points during the study period. Hormonal analysis (cortisol and melatonin) was conducted in six participants over 1–2 consecutive days alongside RNA sampling. PAP (Papanicolaou)-based staining of epithelial cells and leukocytes was performed in three participants concurrently with RNA sampling. Fourteen participants completed the MEQ-SA questionnaire^12^ for chronotype determination (Fig. 1). For saliva volume and RNAprotect ratio optimization, four participants provided samples at a defined time point (13 h). Human, mouse, and baboon datasets (mammalian reference datasets, for details, see “Materials”) were obtained from GEO or ArrayExpress^33–35^ for tissue synchrony analysis of *ARNTL1* and *PER2* and *in-silico* time series analysis to determine the minimum data points required for circadian rhythm assessment.

This study integrates diverse data types for human circadian rhythm assessment (Fig. 1), addressing technical and clinical goals. Firstly, we explored saliva’s feasibility and biological basis for the analysis of the circadian rhythm profile. We aimed to demonstrate (a) saliva’s potential as an optimal starting material, given the known tissue synchronization across clocks within the organism; (b) protocol optimization for high-quality RNA extraction from saliva; and (c) the intrinsic nature of the circadian rhythm, unaffected by cell composition variability. Importantly, saliva’s convenience for home or outpatient sampling is clinically advantageous.

Secondly, we aimed to validate our protocol’s performance and robustness for assessing circadian rhythms via clock gene RNA expression in saliva. Our goals include: (a) demonstrating assessable circadian profiles from clock gene RNA expression; (b) confirming rhythm stability over consecutive days; (c) identifying conserved correlations between circadian parameters in mouse reference profiles and our cohort; and (d) proposing strategies for sample reduction while maintaining analysis quality, beneficial for real-world applications, offering time-saving and flexibility, particularly in hospital settings.

Thirdly, we sought to correlate the obtained gene expression analysis results with established circadian rhythm assessment methods such as melatonin and cortisol hormone levels and chronotype. Clinically, we propose gene expression profiling of core clock genes as valuable for disease prevention, treatment, and diagnosis. This approach can directly link rhythmic information to metabolic networks or drug targets, facilitating personalized medicine through companion (essential for effective personalized medicine) or complementary (provides valuable information, but it is not required for the administration of the drug) diagnostics.

We focused here on specific core clock genes as they are known to steer downstream clock-controlled genes and provide a good estimate of the overall clock status, therefore providing a robust and cost-effective characterization of the expression profile of the peripheral clock^36^. The chosen clock genes *ARNTL1, NR1D1,* and *PER2* were shown in previous studies to exhibit a robust and well-detectable circadian rhythm in saliva and/or oral mucosa^26,28,37^. Depending on the biological context, other clock genes might be meaningful. For instance, *PER3*, as compared to other *PER* isoforms, was shown to have stronger oscillation phenotypes in blood, oral mucosa, and hair follicles regarding the amplitude, inter-individual variability, and significance of rhythmicity^30,38,39^. Our protocol is not limited to the clock genes shown, but can be used for other clock- and clock-controlled genes^26^.

### Saliva is an optimal biological material to access the circadian rhythm in humans

We previously showed the feasibility of assessing circadian rhythms in peripheral clocks in humans using saliva samples^26^. Here, we analyzed circadian profiles of a variety of tissues in publicly available datasets of human, mice and baboon^33–35^. The data showed phase synchronization of clock genes *ARNTL1* and *PER2* across peripheral tissues (Supplementary Fig. 1). This validates the use of peripheral tissues for circadian rhythm analysis, supporting the usage of saliva, with its practicality, ease of handling, and non-invasive collection protocols, as a suitable biological material for assessing circadian rhythms in human studies. We established a protocol for obtaining RNA in sufficient yields and quality to allow for quantification of gene expression from saliva in a previous study^26^. To further optimize the previous protocol, we chose a study subgroup (*n* = 4). We used RNAprotect, a standard preservative to protect RNA from degradation, and analyzed different ratios of saliva:RNAprotect, as well as different volumes of saliva. Following RNA extraction, RNA concentration and quality/purity (as defined by A260/230 and A260/280 values) were determined. Overall, maximal yields could be obtained with a 1:1 ratio and 1.5 mL of saliva. Additionally, no significant variance in RNA quality/purity was observed across the conditions tested (Supplementary Fig. 2). Thus, we used a 1:1 ratio and 1.5 mL saliva for subsequent experiments.

**Fig. 2:**
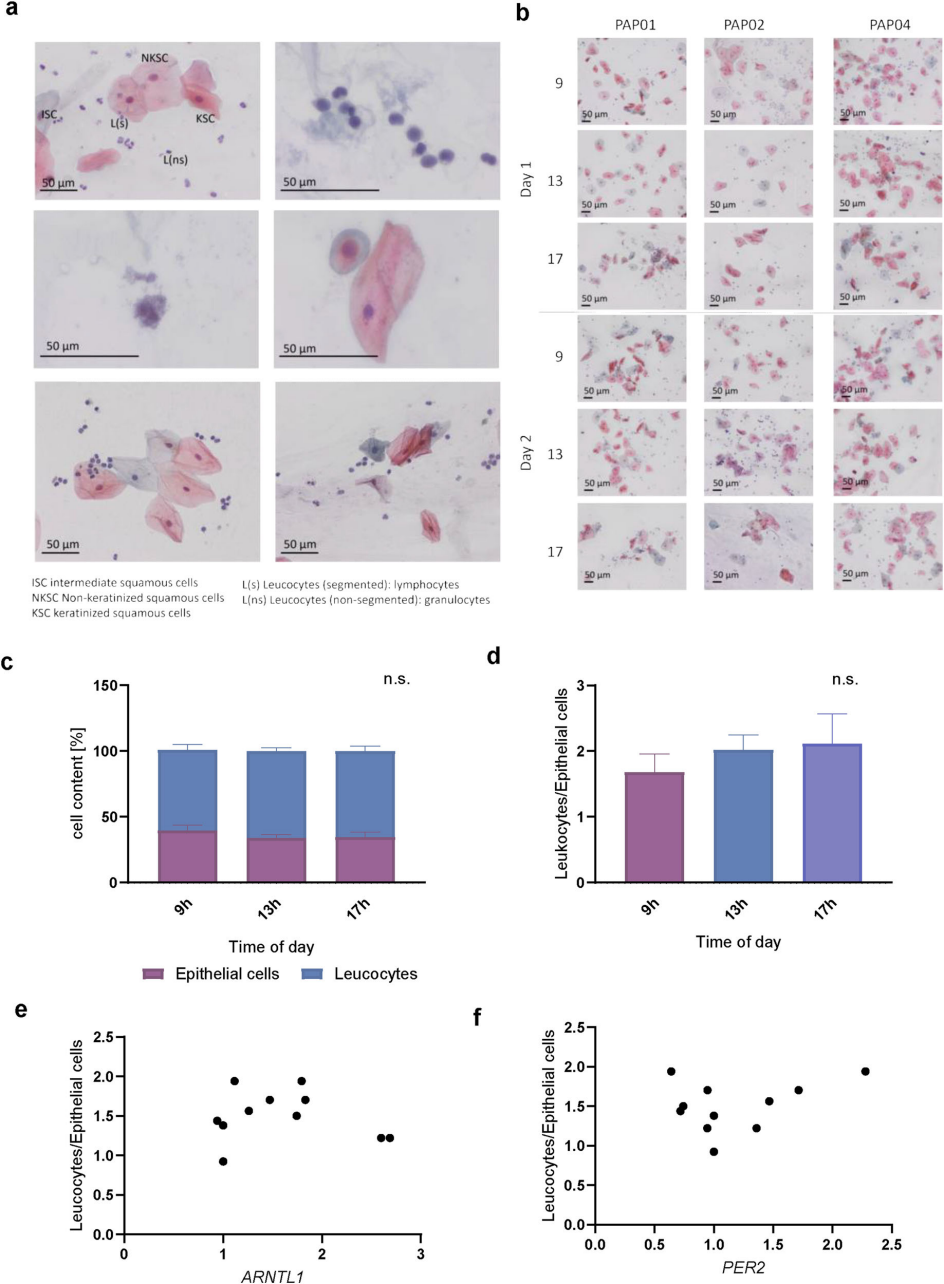
Time-dependency of cell composition in saliva. Three participants provided saliva at 3 timepoints during one day (9 h, 13 h, 17 h), and samples were stained using the PAP method to distinguish between epithelial cells and leukocytes. Epithelial cells and leukocytes were counted from 8 separate fields of the respective slide and the ratio L/E was calculated. **a**, **b** show exemplary pictures of PAP staining. **a** Depicted are the cell types found in saliva and **b** exemplary images for each participant and timepoint. **c**–**e** shows time-dependency in saliva. **c**, **d** show the cell composition at the sampling time points. Day 1 and 2 were summarized. Significances were calculated by one-way ANOVA. **e**, **f** shows the Spearman correlation between clock genes *ARNTL1* or *PER2* and the L/E ratio. *n* = 18. Size bars are 50 µm.

Next, we investigated whether alterations in cellular composition, specifically the leukocyte/epithelial cell (L/E) ratio, might impact the circadian rhythmicity of the core-clock genes measured. For that, we collected the saliva of three participants at 3-time points (9 h, 13 h, 17 h) over 2 days, and, in parallel, assessed the cellular composition, and gene expression of *ARNTL1* and *PER2*. The cells were stained using the PAP method, which showed good preservation of cellular properties and allowed us to distinguish between the two main cell types expected in saliva, epithelial cells, and leukocytes (Fig. 2a, b), as previously described^32,40^. We could also observe bacteria from the oral flora (Fig. 2a, b) on the samples, as expected, which are unlikely to influence the RT-PCR measurements. Firstly, because no bacterial homologs to human clock genes have been found to date^41,42^, secondly, because we used primers validated by the manufacturer to be specific for the human genes analyzed.

The cell composition did not show a significant time-dependency (Fig. 2c, d). Furthermore, the L/E ratio did not significantly correlate with either *ARNTL1* or *PER2* which indicated that the observed diurnal fluctuations of core clock genes in saliva cellular composition were indeed due to internal circadian rhythmicity of the cells rather than fluctuations in cell composition (Fig. 2e, f).

### Gene expression of core clock genes exhibits circadian rhythmicity

**Fig. 3:**
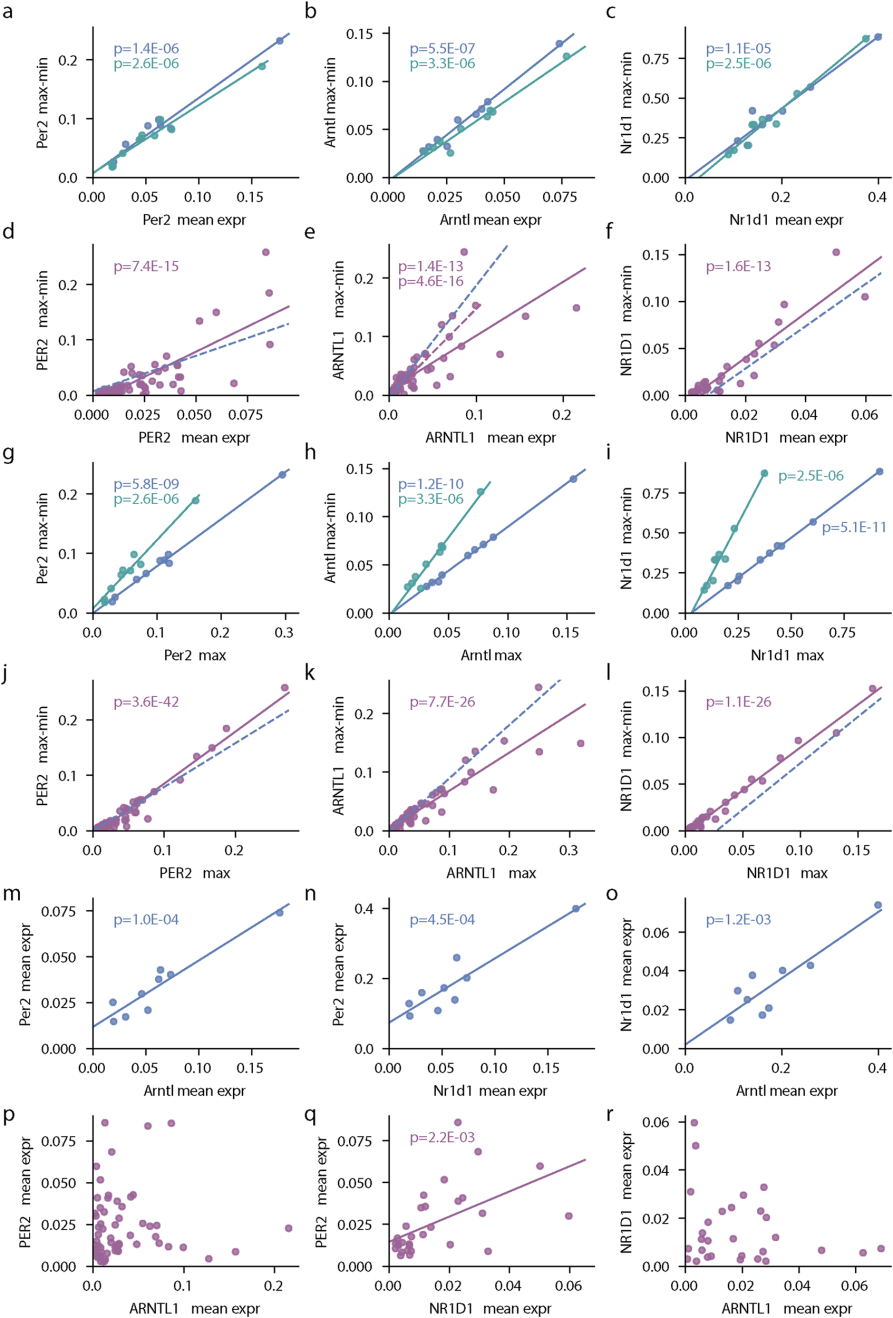
Correlation of different characteristics of the gene expression profiles. Max–min is the difference between maximal and minimal expression values. Dots represent one subject evaluated over one day (analysis assumes independence between days; **d**–**f**, **j**–**l**, **p**–**r**), or nine mouse peripheral tissues (each a 46-h time series; a-c, g-i, m-o). **a**–**f** Circles depict the relation between max-min and mean for *Per2* (**a**) and *PER2* (**d**), *Arntl* (**b**) and *ARNTL1* (**e**), and *Nr1d1* (**c**) and *NR1D1* (**f**). Linear regression suggests a correlation between max-min and mean (*p* < 0.001). Slopes match between human (violet) and mouse (blue, dashed, and straight lines are the same) in **d**, **f**, while for **e**, treating the three largest values of *ARNTL* mean levels as outliers result in a correlation line (dashed violet line) closer to the mouse. The regression slope is lowered when restricting the mouse data to the human interval (green circles and lines), see methods. **g**–**l** Correlation of gene expression amplitude and maximum expression. Circles depict the relation between max-min and max for *Per2* (**g**) and *PER2* (**j**), *Arntl* (**h**) and *ARNTL1* (**k**), and *Nr1d1* (**i**) and *NR1D1* (**l**). Linear regression suggests a correlation between max-min and max (p < 0.001). **m**–**r:** Circles depict the relation between the mean expression of different clock genes. Linear regression suggests a correlation between all mouse genes (**m**–**o**, *p* < 0.002), for humans, mean *NR1D1* expression correlates with mean *PER2* expression (**q**).

For circadian profiles, the difference between maximal and minimal expression value over time (max-min) represents an estimate of the oscillation amplitude. For the peripheral tissues of mice, we observed a correlation between max-min and mean expression level (mean over time) for all core-clock genes (Fig. 3a–c, Supplementary Fig. 3). The correlation was particularly strong for *Per2, Nr1d1*, and *Arntl* (Supplementary Fig. 3), which motivated us to measure these genes. We found a similar correlation between amplitude and mean for the gene expression in human saliva (Fig. 3d–f, full line from human data, dashed line from mouse data). The observed small reduction in slope in the human data as compared to the mouse data could be explained by the limited time period of measurements because restricting the measurements of the mouse data to a similar time period reduces the regression slope (Fig. 3a–c, green line). A correlation between max-min and mean level should imply a correlation of max-min with the maximal expression value (max), which we indeed observe in the human and mouse data (Fig. 3g–l). In this case, the match of the correlation slopes is reduced, potentially because the measurements are based on fewer data points, i.e., evaluation of the highest expression with only 4 data points per day is less robust as compared with the mean level. Similar regression lines for humans and mice suggest that we can indeed access fundamental parameters of circadian profiles, in particular, good estimates of mean expression and expression amplitude, of *PER2* and *ARNTL1* in our saliva-based data.

Core-clock genes interact in a transcription-translation network, and thus we investigated the correlation between the temporal mean level of those different genes. In the mouse data, we found a significant correlation over the different tissues between the expression of all pairs of genes, *Per2* and *Arntl*, *Per2* and *Nr1d1*, and *Nr1d1* and *Arntl* (Fig. 3m–o). In the human data, the mean expression of *PER2* and *NR1D1* was correlated over different subjects and days, while *PER2* and *ARNTL1,* as well as *NR1D1* and *ARNTL1,* were not linearly correlated in our data set (Fig. 3p–r). The correlation in the mouse data might be individual-specific, in which case we would not expect a correlation over different human subjects, or the missing correlation for *ARNTL1* might be explained by a putative stronger influence of lifestyle factors on *ARNTL1* expression as compared to *PER2* and *NR1D1* expression. While the above correlation analysis focuses on the relative size of circadian characteristics between humans and mice, we also find that the circadian profile, when analyzing two consecutive days and taking the mean over many subjects, shows, also in absolute values, a relation to a mouse reference tissue (Supplementary Fig. 4).

**Fig. 4:**
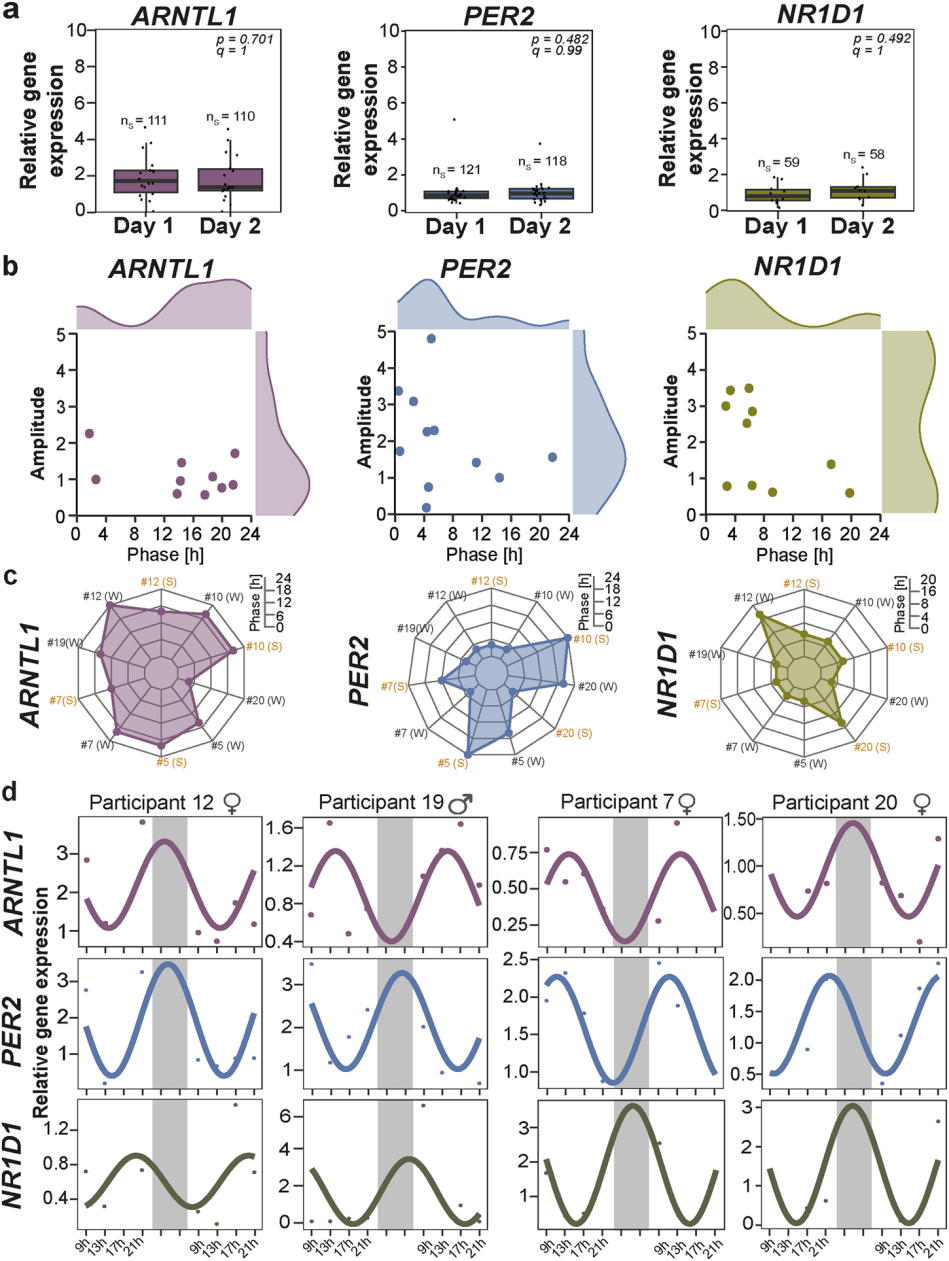
Circadian evaluation of *ARNTL1, PER2*, and *NR1D1* genes measured from the saliva of healthy participants with TimeTeller^®^ kits. **a** Boxplot depicts the reproducibility in gene expression of *ARNTL1*, *PER2*, and *NR1D1* between Day 1 and Day 2. **b** The acrophase plot depicts the distribution of participants according to their phase and amplitude. **c** The radar plot depicts the phase (time of first peak expression) of each participant who carried out saliva sampling at different times of the year (# represents the participant number; W: saliva sampling carried out during winter time; S: saliva sampling carried out during summer time). **d** Representative images of circadian profiles among different participants. The gray shade represents the sleep time (~23 h–7 h) window between the two days of saliva sampling.

Core-clock genes (*ARNTL1, PER2, NR1D1*) exhibit circadian variations throughout the day. We assessed the reproducibility of gene expression measurements in saliva samples across two consecutive days, using TimeTeller^®^ kits, for *ARNTL1*, *PER2*, and *NR1D1*. No significant differences were observed between Day 1 and Day 2 (Fig. 4a; Pairwise Wilcoxon test with Bonferroni correction). Some participants provided saliva samples over four consecutive days, confirming the robustness of our method, but also revealing interindividual variability in circadian parameters and profiles of *ARNTL1, PER2*, and *NR1D1* (Fig. 4d). Despite individual differences, certain similarities emerged; for instance, *ARNTL1* expression peaked after 16 h for most participants (Fig. 4b), while *PER2* and *NR1D1* peaked before 12 h. Additionally, saliva sampling at different times of the year showed changes in peak expression for *ARNTL1, PER2*, and *NR1D1* in some participants (Fig. 4c). Seasonal variations, particularly in daylight duration, can directly impact individual circadian rhythms, as also previously reported^43^. Male and female circadian rhythms differ in key aspects, such as cycle length, hormonal regulation, and sensitivity to environmental cues. Although the primary focus of this study was not to investigate sex differences—an analysis that would require age-matched participants—we did observe trends suggesting differences among male as compared to female participants in terms of mean gene expression, phase, and amplitude of circadian rhythms. While these differences were not statistically significant, likely due to the small sample size and confounding factors like age (Supplementary Fig. 5), they confirm sex-related variability in circadian regulation as previously reported^33^.

For our analysis, we primarily utilized 8 saliva samples collected during the active phase of an individual over two consecutive days, from 9 h to 21 h. However, reducing the number of sampling time points for circadian rhythm assessment would enhance feasibility, streamline sampling, and reduce analysis costs. Such reduction is anticipated to improve compliance and better align sampling with the daily schedule of participants or the clinical routines, and thus it is relevant for routine measurements. To explore this, we employed a newly developed algorithm on published datasets from SW480 cells^44^ and the Gtex dataset from healthy participants (transverse and sigmoid colon tissues), extrapolating circadian profiles from single-time-point samples^33^, and compared circadian gene expression for *ARNTL1* and *PER2* gene expression. Results indicated aligned circadian expression (Supplementary Fig. 6a). Additionally, using RNA-Seq data from SW480 cells collected every 3 h over 30 h, we tested various *in-silico* conditions for sampling interval and frequency. Our findings suggest successful circadian rhythm assessment with at least 4 data points within a day (Supplementary Fig. 6b).

### Circadian expression profiles of core clock genes correlate with daily hormonal variations

**Fig. 5:**
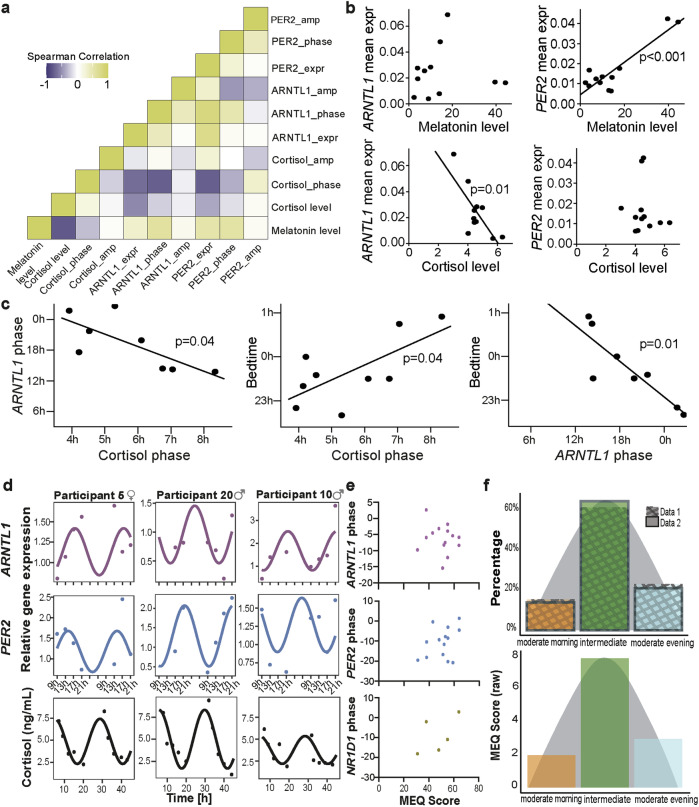
Correlation of the clock gene expression with hormonal levels and MEQ scores. **a** correlation heatmap plot represents the positive/negative correlation between the hormonal and gene expression data. **b**, **c** scatter plots depict the relations between *ARNTL1/PER2* mean expression or phase with hormonal data. **d** Representative circadian profiles of *ARNTL1*, *PER2*, and cortisol among three participants. **e** The Pearson correlation between clock gene expression and MEQ scores is presented. All gene expression values for each participant were summarized for analysis (*n* = 19 for *ARNTL1* and *PER2*, and *n* = 10 for *NR1D1*). **f** the distribution of MEQ results is depicted as a percentage to standardize results from different population sizes (upper panel) and in raw MEQ values in Data 1 (lower panel). Data 1 includes MEQ results from Basti et al.^26^ and newly generated data in this study (*n* = 13), while Data 2 was retrieved from Randler et al.^45^.

To evaluate the reproducibility of *ARNTL1*, *PER2*, and *NR1D1* gene expression data, we calculated several error metrics: mean absolute error (MAE), mean squared error (MSE), root mean squared error (RMSE), and mean bias error (MBE). The MAE was 0.112, indicating an average deviation of 0.112 units between Day 1 and Day 2 expression values. The MSE was 0.021, reflecting the average squared difference, with lower values signifying better consistency. The RMSE was 0.1475, representing the typical error magnitude in the same units as the expression data. The MBE was 0.049, suggesting a slight positive bias, with Day 2 expression values slightly higher than those on Day 1. Overall, these metrics demonstrate strong reproducibility with low errors, confirming consistency in the gene expression data across days. For a more comprehensive assessment of the participants’ circadian profile, we aimed to relate the gene expression profiles determined to other methods reflecting different layers of circadian regulation, i.e., the hormones cortisol and melatonin^2^, and chronotype assessment using the MEQ-questionnaire^12^, which we measured in parallel. Spearman correlation analysis was carried out between *ARNTL1*, *PER2*, and cortisol circadian parameters (phase, amplitude), as well as between their mean levels (Fig. 5a). Cortisol mean level showed a significant negative correlation with melatonin level (*r* = −0.78, *p* = 0.04). Cortisol phase also showed a significant negative correlation with *ARNTL1* phase (*r* = −0.75, *p* = 0.05) and *PER2* mean expression (*r* = −0.75, *p* = 0.05; Fig. 5a). In addition, MI and MIC analyses helped in deciphering the linear and non-linear relationship between gene expression and hormonal data (Supplementary Fig. 7). MI measures the dependency between two variables, i.e., MI of 0 indicates that the variables are independent of each other and higher values indicate a stronger dependency. MIC identifies the strength of the association between the two variables, and its value closer to 1 indicates a strong association. Both MI and MIC showed that *ARNTL1* expression (MI = 0.45, MIC = 0.52) and *ARNTL1* phase (MI = 0.73, MIC = 0.98) had a strong relation with Cortisol phase (Supplementary Fig. 7).

We did not determine circadian profiles for melatonin as it was only measured at one timepoint per day, but found a correlation of melatonin measured at bed-time with the mean gene expression of *PER2*, but not with the mean gene expression of *ARNTL1* (Fig. 5b). Interestingly, we found a correlation of the mean cortisol level with *ARNTL1*, but not with *PER2* (Fig. 5b). We also found correlations between the acrophase of *ARNTL1* and the acrophase of cortisol (Fig. 5c). Furthermore, both acrophases correlate with the bedtime of the subjects at the sampling day (Fig. 5c). The correlation of *ARNTL1* with cortisol might hint at a stronger dependence of *ARNTL1* on the particularities of daily life, such as the amount of stressor exposure as measured by the “stress hormone” cortisol. *PER2* and melatonin, on the other hand, might both be correlated positively as both depend strongly on light exposure. Moreover, the circadian profile of *ARNTL1*, *PER2*, and cortisol depicted variation among different participants (Fig. 5d). Cortisol showed good rhythmicity in our participants (see Fig. 5d for representative examples).

Finally, we compared the circadian phases of *ARNTL1* and *PER2* with the MEQ scores of the participants. As mentioned in the introduction section, the MEQ score is seen as a rough estimate of the circadian phase^11^. For our participants, we did not find a significant correlation between gene expression and MEQ score (Fig. 5e). The distribution of MEQ scores on our dataset (*n* = 14) seems to represent the same distribution as reported from a larger population study (*n* = 594) (Fig. 5f). This data was retrieved from Randler et al., the German version of the reduced Morningness-Eveningness Questionnaire (rMEQ)^45^. Since the rMEQ correlates well with MEQ^46^, and the study originates from a distribution in Germany, this data represents an appropriate reference. As expected, most participants showed an intermediate chronotype (Fig. 5f). In both populations, extreme chronotypes were not observed.

Altogether our results showed the feasibility and robustness of our protocol to measure the circadian rhythm via clock gene expression in saliva, which can be used in a health and disease context.

## Discussion

The proper functioning of the circadian clock is strongly linked to health, and its dysregulation is associated with pathological phenotypes. A disruption of the circadian rhythm e.g., by shift work or artificial light at night^1,4–8,47^ can result in a variety of diseases including circadian rhythm sleep-wake disorders as described in the ICD-11 guidelines^48^. Characterizing the individual circadian rhythm profile (including amplitude, acrophase, and MESOR/mean expression) is crucial, serving as a potential companion biomarker for assessing a person’s health status, for disease prevention and lifestyle optimization, and for facilitating personalized circadian-based therapy (chronotherapy)^18^.

There are several examples where the circadian rhythm of clock genes in saliva was reported to be linked to disease. A study published recently showed a disturbance of rhythmicity in children with FASD (Fetal Alcohol Spectrum Disorder) compared to healthy children, which correlates well with their disturbed sleep patterns^49^. FASD was reported to affect up to 1% of children^50^, a significant percentage of the population, and early diagnosis was shown to be critical for optimal treatment^51^. FASD lacks a clear gold standard of diagnosis to date and can be challenging and complex to diagnose especially in children showing less severe phenotypes, therefore the diagnosis of circadian profile disruption in saliva may be an appropriate method to use in initial routine diagnostics of FASD^51^. Another recent study showed lower levels of *ARNTL1* in the saliva of obese men, which could be linked to altered levels of metabolic markers in the blood^52^. Given the easier and non-invasive sampling of saliva compared to blood and apparently the reliance on a single marker for diagnosis, our method may provide a good estimate of the metabolic health of an individual. As metabolic syndrome has been on the rise for years^53^ efficient diagnosis of metabolic disorders will only become more important in the future. Other examples that are closely linked to salivary peripheral circadian rhythms are oral diseases such as Head-and-Neck cancer and Sjörgens Syndrome, which were shown to display altered circadian profiles in oral mucosa^54^.

In our study, we demonstrated the robustness and applicability of our TimeTeller^®^ approach for measuring the circadian rhythm in healthy participants using relative RNA expression of core-clock genes in saliva. Additionally, we found no correlation between circadian gene expression and saliva cell composition (epithelial cells, leukocytes), indicating that gene expression fluctuations are tied to internal circadian rhythms rather than variations in cell composition. Our findings on saliva cell content (36% epithelial cells) align with previous studies in adults (47% and 42%), despite our small sample size. Interestingly, the proportion is notably higher in children (70%)^31,32^. We found no significant correlation between circadian gene expression and cellular composition, which indicates that variations in cell composition do not steer the here reported rhythmicity of clock genes. The oral immune system was observed to show diurnal variations in terms of cytokines and eicosanoids^55–59^. These mediators tended to peak in the morning. On the other hand, in oral mucosa, biopsies of six individuals p53 protein levels peaked in the morning, whereas cyclins peaked throughout the day, reflecting the progression from G1/S to M-phase of the cell cycle and Ki-67 peaked at night^60^. Thus, it seems that while oral mucosa epithelial cells exhibited diurnal variations, saliva samples did not. Hence, saliva may offer a more suitable substrate for analyzing the circadian rhythmicity of clock genes compared to oral mucosa samples, such as buccal swabs. We found microbiota in the PAP stains as expected which exhibited inter-individual and temporal variations. The oral microbiome consists of over 700 species and is known to exhibit close crosstalk with the squamous epithelium of the oral cavity^61,62^. Several studies analyzed the composition of the oral microbiome with regard to the circadian clock^58,63–66^. Five studies showed significant rhythmicity in specific bacterial genera with only limited overlap between the studies^58,63–66^. This is most likely due to small study numbers, different methodologies and levels of control of the environment, and high interindividual differences in the microbiome^66^. Incubating saliva in vitro completely abolished microbial rhythms suggesting that they were controlled by the host environmental conditions^66^. Although we did not analyze the microbiota on our stains in more detail histologically, they morphologically resembled *Streptococcus*, and we also observed time-dependent effects in the abundance of this bacterial species, which would be in line with the previous studies^63,65,66^. Given the importance of the oral microbiota in oral homeostasis^62^ it would be interesting to look at this population closely in future studies. We used primers that were bioinformatically verified by the manufacturer to be specific for humans; therefore, we are confident that the rhythm we detected on the RNA level stems from human cells and not from variations in the oral microbiome.

Our analysis of the circadian profiles of mammals over different tissues showed tissue synchronization as expected^2^, which strengthens the idea of using peripheral clocks, e.g., in saliva due to its convenience for sampling, as a good representation of the overall circadian rhythm of an individual. Especially in the context of a broader application in both optimization of daily routines as well as in clinical settings, saliva offers the advantage of non-invasiveness, and easy collection protocol that can be applied in an at-home setting. In previous studies, the feasibility of assessing the circadian rhythmicity of core clock genes in saliva was shown in small cohorts^26,28^. To our knowledge, our study is the first aiming to combine such a comprehensive set of data displaying a variety of facets of circadian rhythmicity.

Focusing on future applicability, circadian profiling of clock genes, as demonstrated here, emerges as a pivotal component in disease treatment and prevention strategies that incorporate circadian rhythm considerations. Numerous studies underscore the significant role of core clock genes across diverse physiological processes, frequently altered in conditions such as cancer and inflammatory disorders^1,3–7,41^. Therefore, understanding the profile of these clock genes can furnish invaluable insights across various contexts, potentially surpassing other methods of circadian profile analysis. Due to the phase correlation between *ARNTL1*, cortisol, and bedtime (Fig. 5c), circadian profiles of gene expression might allow for accessing an internal phase of a subject while measuring only a few genes.

In our analysis, we observed significant interindividual variability in participants’ circadian profiles, consistent with prior research^26,28,29^. This underscores the importance of directly measuring circadian clock profiles rather than relying on rough estimates based on wake-up times. Clinical studies relying on such rough estimations often led to conflicting results, for example, differences in outcome dependent on sex or age^18^. We noted a trend suggesting that *ARNTL1* may be more susceptible to changes in daily routines than other clock genes. This finding aligns with numerous studies reporting the influence of lifestyle factors, such as feeding/fasting times and exercise, on *ARNTL1*^67,68^, but further comprehensive study designs are necessary to confirm this trend.

To accurately determine a circadian profile, it is relevant to define the frequency of sampling needed within 24 h. In a study by Akashi et al. using three or four data points over time was sufficient to accurately assess the circadian rhythms with 3-h or 6-h sampling frequency, but not with longer sampling intervals^30^. Our data supported the notion that circadian profiling using 4 data points over 24 h or spreading the data collection over 48 h, but with five data points was possible using the genes *ARNTL1* and *PER2*. We found multiple possible combinations that could be fitted into a normal wake-sleep cycle. A reduced-timepoint-a-day sampling protocol is beneficial to simplify sampling, reduce costs, and improve compliance.

For our group of healthy individuals, we measured a positive correlation between mean and amplitude of the genetic profiles. Given the strong correlation in mouse data, the ratio of amplitude and mean, i.e., the amplitude of mean-normalized oscillations, might be useful as an interesting summary marker for circadian rhythms in future clinical studies, and as a base line comparison for detecting individual circadian dysregulations.

We found a positive correlation of *PER2* mean expression with melatonin and a negative correlation of *ARNTL1* mean expression and phase with cortisol. As *PER2* expression is suppressed by light, this correlation was expected^2^. Nováková et al. found a positive correlation of melatonin with *PER1* and a trend towards a positive correlation of melatonin with *PER2* though not significant^28^. This is generally in line with our results. Yurtsever et al.^29^ did not find a correlation between cortisol and *PER* expression, which is again in line with our data. *ARNTL1* was linked to inflammation in several previous publications^69–71^. Due to the role of cortisol in inflammation suppression^72^, a negative correlation with *ARNTL1* was therefore not unexpected. The MEQ score is generally seen as a correlate of the circadian phase, however, it is subjective and biased towards sleep onset and wake-up times. Furthermore, it was developed based on the behavioral patterns of a homogenous group of students and may therefore have weaknesses in applying it to a more heterogenous population as explored on in greater detail in the introduction^11^. We found that the MEQ distribution in our study participants reflected well the distribution found in a larger German cohort^45^, chosen for comparison to reduce geographical bias. Most subjects fell into the intermediate MEQ range. We did not find a significant correlation between MEQ score and either expression of *ARNTL1*, *PER2*, or *NR1D1*. Previous data found that MEQ scores generally related well to CBT, melatonin onset, and cortisol; however, not in all cases^11^. For instance, Duffy et al. found a correlation between the CBT phase and MEQ score only in young, but not in older adults^73^. As explained above, our methodology could be especially useful in the detection of diseases linked to disruption of the circadian rhythm at the gene expression level which may not necessarily be that well correlated to melatonin/cortisol^54^. It is not the aim of our method to replace either hormones or any type of subjective questionnaires but rather to provide a means to characterize the profile of the core clock, represented by the genes measured in a non-invasive and user-friendly way. Therefore, our approach for profiling circadian rhythms with the TimeTeller^®^ methodology should not be seen as a substitute for the above-mentioned methods, but rather as an additional analysis which provides comprehensive information on the circadian rhythm of a given person, at different levels, and includes the amplitude, the phase, the mesor and the period of the oscillations. This is particularly useful if the goal is indeed to characterize the profile of the molecular clock. We are currently working on larger clinical studies in cancer, Parkinson's disease, and overall health maintenance, which will define more precisely the scenarios in which our methodology will be particularly useful^74–77^. Having shown that we can robustly measure profiles of circadian core clock genes in saliva with TimeTeller^®^, we do see the potential to apply our protocol and include profiles of additional disease-specific genes for certain pathologies, which will strongly widen the information content by supplementing core-clock profiles with circadian profiles of disease-relevant markers.

Of notice, and in contrast to the in-vivo animal studies performed under controlled conditions (e.g., Mure et al.^78^, Zhang et al.^79^), our study aimed to assess circadian gene expression in human participants following their regular daily routines, using a fully non-invasive and user friendly procedure. This approach introduces variability due to the diverse environmental and lifestyle factors influencing human circadian rhythms, such as light exposure, sleep patterns, and activity levels. These variations, including larger phase shifts between seasons and differences in the expression of genes like *ARNTL1* and *PER2*, are expected outcomes of natural human diversity. Importantly, the observed variability is not a limitation of our method but rather reflects the real-world complexity of circadian behavior, which is crucial for translating these findings into clinical settings. Moreover, repeated measurements on consecutive days showed no significant differences, demonstrating the robustness of our method in profiling circadian rhythms in saliva. The number of samples collected may influence the precision of our method, however as shown by Akashi et al.^30^ and also by our own analysis (Supplementary Fig. 6), it is possible to determine the circadian profile without requiring night-time sample collection, and without significantly influencing the determination of the circadian parameters (Supplementary Fig. 6b). Interestingly, other studies using more frequent sampling showed that circadian genes could show the expected anti-phase relationship of *ARNTL1* and *PER2/3* in some individuals, but not in others. In particular, *ARNTL1* and *PER2/3* were found to be in phase with each other in individuals with sleep disorders^38,80^. Thus, the variability of phenotypes may represent the diversity of circadian behavior, but may also correlate with a particular pathological situation. Such results highlight the need to analyze circadian rhythms and determine their full 24-hour profile. Yet, to be able to define a normal range of circadian phenotypic variations, more data across large population groups is needed.

Despite our study’s limited sample size, we integrate multiple datasets to elucidate the intricate interactions within the circadian rhythm components (clock genes, hormones, cells). Our TimeTeller^®^ method thus allows for the easy generation of profiles of the circadian clock in a complete, non-invasive, and user-friendly way and sets the basis for follow-up studies aiming at characterizing circadian biomarkers in larger cohorts, being one of such biomarkers a full profile of the circadian clock and not merely the circadian phase. Future validation in larger cohorts is planned. Given the significance of salivary biomarkers for diagnostics, we intend to apply these procedures in diverse treatment and prevention studies, leveraging circadian rhythms as a complementary diagnostic optimization tool.

## Methods

### Participants and sample collection

The data was collected between 2018 and 2023 (but not during the COVID high infection time in Germany) and consisted of 21 healthy participants with a mean age of 31 years (25–43 years) and 52.4% females. The individual experiments included 4 to 19 participants, as indicated in the results section. All participants provided saliva at 3-4 timepoints/day over 2 consecutive days for RNA extraction and gene expression analysis of core clock genes, however for participant 18, the RNA concentration was too low for gene expression analysis. Some participants provided saliva for RNA extraction more than once across the year. Six participants provided samples for hormonal (cortisol and melatonin) analysis over one or two consecutive days. Three participants gave samples for PAP-based staining of epithelial cells and leukocytes in saliva. The sampling for the hormonal and cellular measurements was performed in parallel to that for RNA extraction and gene expression analysis. In parallel, each participant filled out the Morningness-Eveningness-Questionnaire-Self Assessment (MEQ-SA) questionnaire for chronotype determination^12^. For the optimization of saliva volume and saliva/RNAprotect ratio for RNA extraction a total of four participants provided samples at a defined timepoint (13 h) to avoid possible time-of-day dependent fluctuations of saliva composition and RNA amount in saliva.

### Ethics statement

All procedures involving human subjects were approved by the Charité—Universitätsmedizin Berlin Ethics Committee (EA2/188/18; EA4/040/20). The participants were enrolled on a voluntary basis and signed the informed consent approving the analysis of their data in the scope of the study.

### Sample collection

Unstimulated saliva samples were collected using TimeTeller^®^ Kits at 3-4 timepoints (9 h, 13 h, 17 h, 21 h) for 2 consecutive days. One hour before sample collection participants refrained from eating, drinking, smoking, and oral hygiene. Saliva was collected in 3 mL collection tubes with RNAprotect Cell reagent (Cat No. 76526, Qiagen), the tube was shaken vigorously to mix and stored at 4 °C for up to 1 month or at -20 °C for long-term storage. For ratio optimization, the ratios saliva:RNAprotect 1:1 @ 1.5 mL saliva, 1:2 @ 1 mL saliva, 2:1 @ 1 mL saliva, and 1:2 @ 0.5 mL saliva were tested. For all other experiments, the ratio was 1:1 (1.5 mL saliva and 1.5 mL RNAprotect).

### Cortisol and melatonin

The participants who gave consent for the collection of their hormonal levels were provided with a commercially available kit for Cortisol and Melatonin analysis from Cerascreen®. For cortisol measurements, saliva was sampled directly after and 0.5 h, 1 h, 2 h, 5 h, 8 h and 12 h upon awakening. For melatonin quantification, saliva was sampled 30 min before going to bed.

### RNA extraction and quantification

RNA was extracted as previously described using TimeTeller® Kits according to the manufacturer indications^9,26^. Gene expression fold-change was calculated using the ΔΔCT method normalizing CT values first to *GAPDH* and then to mean expression value of the respective target gene. The procedures have been described elsewhere in detail^26^.

### PAP staining and microscopy

50 µL saliva was pipetted on a glass microscopy slide and immediately fixed using RotiFix spray (Cat No. CL85.1, Roth, Karlsruhe, Germany). After drying the fixing spray, the slides were further processed.

For PAP staining the PAP rapid stain kit (Cat No. 9195.1, Roth, Karlsruhe, Germany) was used according to the instructions of the manufacturer. Briefly, unstimulated saliva was collected for 2 consecutive days at 9 h, 13 h, and 17 h 1.5 mL saliva was mixed with 1.5 mL RNA protection for gene expression analysis as described above. 50 µL saliva was spread on a glass microscopy slide and fixed using RotiFix Spray. After drying, the slides were incubated in a descending ethanol series (96%, 80%, 70%; 1 h each). Afterward, slides were incubated in solution 1 (modified hematoxylin solution; 3 min), blued in tap water for 3 min, and incubated in an ascending ethanol series (70%, 80%, 90%; 30 s each). Slides were then incubated in solution 2 (modified polychrome solution), rinsed in 96% ethanol, and dried in 96% ethanol (2 × 30 s) and 100% ethanol (3 × 30 s). Roticlear (Cat. No A538.1, Roth, Karlsruhe, Germany) was used as Intermedium (2 × 1 min, 1 × 3 min), and slides were mounted in RotiMount (Cat. No HP68.1, Roth, Karlsruhe, Germany) and coversliped. After hardening of the mounting medium slides were stored at 4 °C.

### Rhythmicity analysis

Gene expression data was processed using the multivariate harmonic regression model *E*_gc_ = μg + a g cos (φ c) + bg sin (φc) + *ε*, where “*c*” represents the condition, “g” represents the gene, and “*ε*” denotes the Gaussian noise represent random variability in the gene expression measurements as also previously used^33^. The model fits a periodic function (using cosine and sine terms) to the gene expression data, accounting for a 24-h cycle (a fixed period of 24 h used in our analysis). Likelihood Ratio Test is then used to compare the two models (with and without periodic terms) to estimate amplitude and phase. The same procedure was used for the RNA-seq and microarray datasets analyzed. Phase is a circular variable, with phases before and after midnight close in time.

For cortisol, we used a harmonic regression with soft L1 cost, fitting μ + a cos (φ) + b sin (φ) to the cortisol measures, using the Python function scipy.optimize.least_squares(*error*, x0 = [*a* = −0.31084448, *b* = 2.33838041, *μ* = 6.33268443], loss = ‘soft_l1’, f_scale = 0.1) with *error* the absolute difference between experimental data and fit.

### Processing of microarray and RNA-seq transcriptomic datasets

For the mouse microarray dataset (GSE54650) from Zhang et al.^34^, publicly available data was retrieved from the Gene Expression Omnibus (GEO) database and pre-processed using the Robust Multichip Average (RMA) pipeline as described^81^. The baboon dataset (GSE98965) was downloaded from the European Nucleotide Archive (ENA), and SW480 and SW620 RNAseq data is available under the ArrayExpress database under the accession number E-MTAB-7779^44^. RNA-sequencing reads from both baboon and cell line data were aligned to reference genomes, using STAR aligner with default parameters^81^. Gene summarized counts were then normalized using the trimmed mean of M-values (TMM) method, and counts were log2-transformed to the counts per million (CPM) scale. A low gene expression filter of at least 0.5 CPM on average over the entire time series (per tissue) was applied before downstream analysis.

### Comparison with mammalian circadian rhythms

For a direct comparison with our human data, we loaded the series matrix file of the mouse microarray dataset (GSE54650) from Zhang et al.^33^. The microarray expression values of *Arntl*, *Per2,* and *Nr1d1* were normalized at each timepoint by the expression value of *Gapdh*, the housekeeping gene used for the qPCR analysis, which allows us to compare the absolute values of microarray data and qPCR data.

For the correlation analysis of the mice data (Fig. 3, blue), we evaluated the *Gapdh*-normalized data by calculating the amplitude as the difference between minimal and maximal expression value, the maximum expression as the maximal value, and the mean level as the mean overall values over the two days for each peripheral tissue (aorta, adrenal gland, brown fat (anterior dorsum adipose), heart, kidney, liver, lung, skeletal muscle (*gastrocnemius*), and white fat (epididymal adipose)). For the analysis restricted to the human sampling interval (Fig. 3, green), we evaluated the same for the data measured at 4h-14h and 26h-38h since wakeup (see Supplementary Fig. 4). For the correlation analysis of the human data (Fig. 3, violet), we evaluated the gene expression profiles for each subject and day separately, assuming statistically independent measurements for each day for the linear regression analysis. For each set of up to 4 sampling values per day, we calculated the amplitude as the difference between minimal and maximal expression values, the maximum expression as the maximal value, and the mean level as the mean overall values associated with this day and subject.

For Supplementary Fig. 4, we plotted data from different subjects for two consecutive days. While some subjects contributed multiple pairs of days, the calculation of the standard error of the mean (SEM) assumes statistical independence between each pair of days. We excluded data with two or less datapoints over the two days. For Fig. 5, hormonal values for cortisol that were reported as “<2” were plotted for representative purposes as 1, and one melatonin value reported as “>100” was omitted from the analysis.

### Correlation analysis

Pairwise Spearman correlation was carried out between *ARNTL1*, *PER2*, and Cortisol circadian properties (amplitude, phase), melatonin and cortisol levels, and mean expression (2^dcq^) using R. For Fig. 3 and Fig. 5b, c, we calculated the regression line with scipy.stats.linregress() with standard parameters. Reported are the two-sided *p*-values of this function. To analyze the linear and non-linear relationship between gene expression data (*ARNTL1*, *PER2*) and hormonal data, we carried out mutual information (MI) and maximal information coefficient (MIC) analysis using “infotheo” and “minerva” packages in R.

### Statistical analysis

Wilcoxon paired test followed by Bonferroni’s correction was carried out to evaluate the data reproducibility between the two days of saliva sampling. To assess the robustness of our method, statistical error metrics such as mean absolute error (MAE), mean squared error (MSE), root mean squared error (RMSE), and mean bias error (MBE) were carried out. MAE measures the average magnitude of the errors between Day 1 and Day 2, without considering their direction (i.e., whether the errors are positive or negative). MSE is the average of the squared differences between Day 1 and Day 2. It gives more weight to larger differences because the errors are squared before averaging. RMSE is the square root of the MSE and gives an error value in the same units as the gene expression data. MBE shows the direction of the error, indicating whether, on average, the values from Day 2 are higher or lower than those from Day 1. The gene expression data from Day 1 was used as a “predicted” value and from Day 2 as an “observed” value to calculate the error metrics.

$${MAE}=\frac{1}{n}\mathop{\sum }\limits_{i=1}^{n}\left|{{Gene\; expression\; Day}2}_{i}-\,{{Gene\; expression\; Day}1}_{i}\right|$$

$${MSE}=\frac{1}{n}\mathop{\sum }\limits_{i=1}^{n}{\left({{Gene\; expression\; Day}2}_{i}-{{Gene\; expression\; Day}1}_{i}\right)}^{2}$$

$${RMSE}=\sqrt{\frac{1}{n}\mathop{\sum }\limits_{i=1}^{n}{\left({{Gene\; expression\; Day}2}_{i}-{{Gene\; expression\; Day}1}_{i}\right)}^{2}}$$

$${MBE}=\frac{1}{n}\mathop{\sum }\limits_{i=1}^{n}({{Gene\; expression\; Day}2}_{i}-\,{{Gene\; expression\; Day}1}_{i})$$

## Supplementary information

Supplementary Information

## Data Availability

Data is provided within the manuscript, or the supplementary information, or can be obtained upon request from the corresponding author, and following the rules of the ethics procedures of our university.
