# Supplementary material for: Comprehensive integrative analysis of circadian rhythms in human saliva
Source: NPJ Biol Timing Sleep. 2025 May 10;2:17. doi: 10.1038/s44323-025-00035-3 (PMC12912387; doi:10.1038/s44323-025-00035-3)
Supplement: Supplementary file 1 — Supplementary Information [file 44323_2025_35_MOESM1_ESM.pdf]

Supplementary information for:

Integrative Analysis of Circadian Rhythms in Human Saliva: A Comprehensive Approach

Nina Nelson[1], Deeksha Malhan[1], Janina Hesse[1,2], Ouda Aboumanify[3], Müge Yalçin[1,3,4],Georg Lüers[5], Angela Relógio[1,3,4*]

[1] Institute for Systems Medicine and Faculty of Human Medicine, MSH Medical School Hamburg, Hamburg, Germany

[2] Leibniz Institute for Resilience Research (LIR), Mainz, Germany and Johannes Gutenberg University Medical Center, Mainz, Germany

[3] Molecular Cancer Research Center (MKFZ), Medical Department of Haematology, Oncology, and Tumor Immunology, Charité - Universitätsmedizin Berlin, corporate member of Freie Universität Berlin Humboldt - Universität zu Berlin, and Berlin Institute of Health, Berlin, Germany

[4] Institute for Theoretical Biology (ITB), Charité - Universitätsmedizin Berlin, corporate member of Freie Universität Berlin, Humboldt - Universität zu Berlin, and Berlin Institute of Health, Berlin, Germany

[5] Faculty of Human Medicine, MSH Medical School Hamburg, Hamburg, Germany

*Corresponding author: angela.relogio@medicalschool-hamburg.de

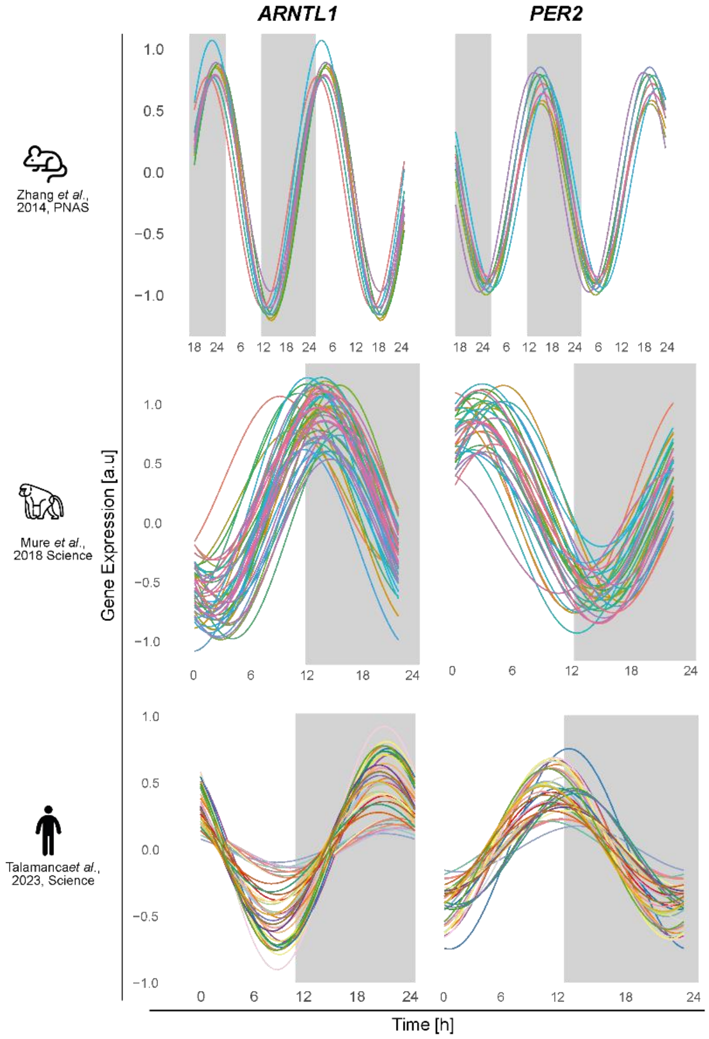

Supplementary Fig. 1: Tissue synchronization in mice, baboon and humans. Depicted are significant circadian expressed genes (q < 0.05) from different tissues using harmonic regression with a fixed period of 24-hours. Mouse data (GSE54651) was obtained from Zhang *et al.*[1] ; baboon data (GSE98965) from Mure *et al.*[2] and human data from Talamanca *et al.*[3] (Age range 21-70 years; 33% females and 67% males). Mouse data was collected from 12 tissues, 48 hours, every 2 hours whereas baboon data included 64 tissues collected over 24 hours, every 2 hours. Human data included n=914 donors, 46 tissues (single time point experiment). The curves were extrapolated based on Circular Hierarchical Reconstruction Algorithm (CHIRAL)[3].

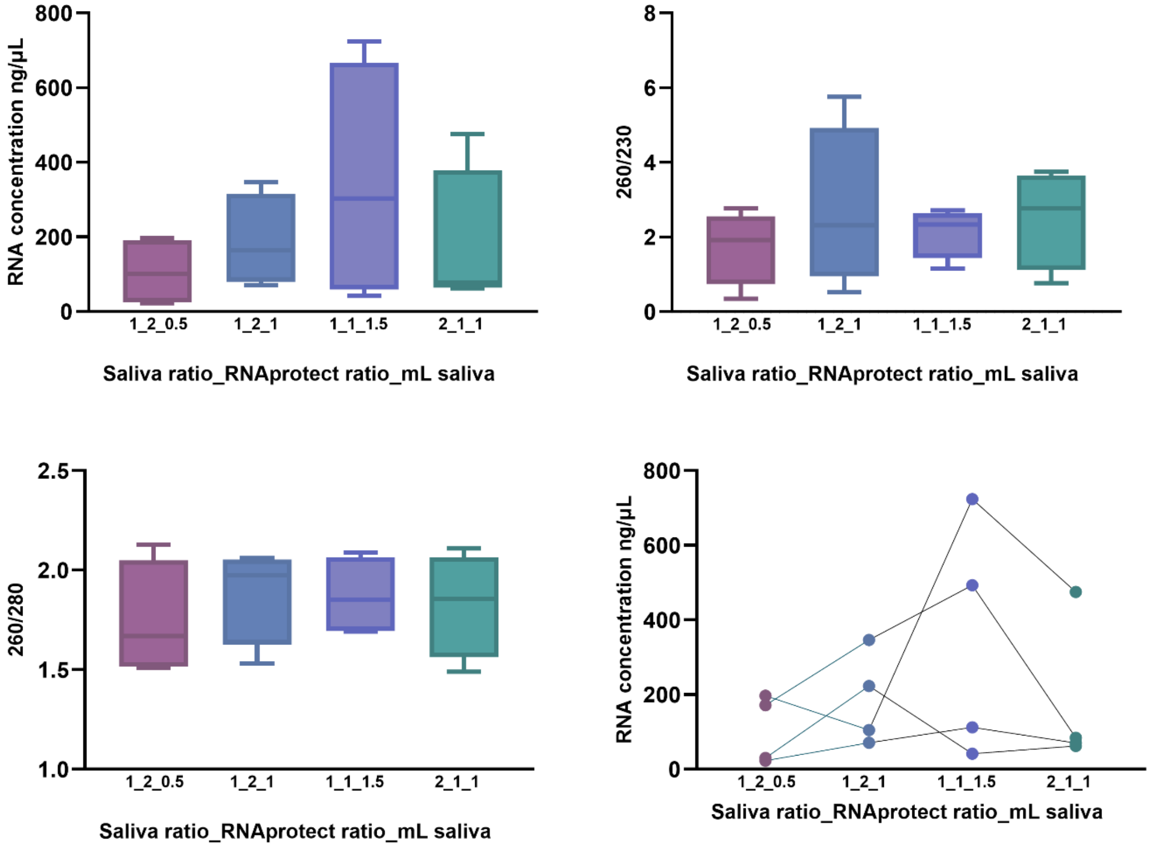

Supplementary Fig. 2: Optimization of salivavolume vs RNAprotect ratio. For a set of four participants saliva was collected at the same timepoint. Different conditions of varying saliva volume/RNAprotect volume ratio were tested in order to identify the optimal sampling protocol. RNA was extracted and RNA concentration, as well as the ratios 260/230 and 260/280 were measured to assess RNA purity. The conditions were compared by one-way ANOVA, n=16.

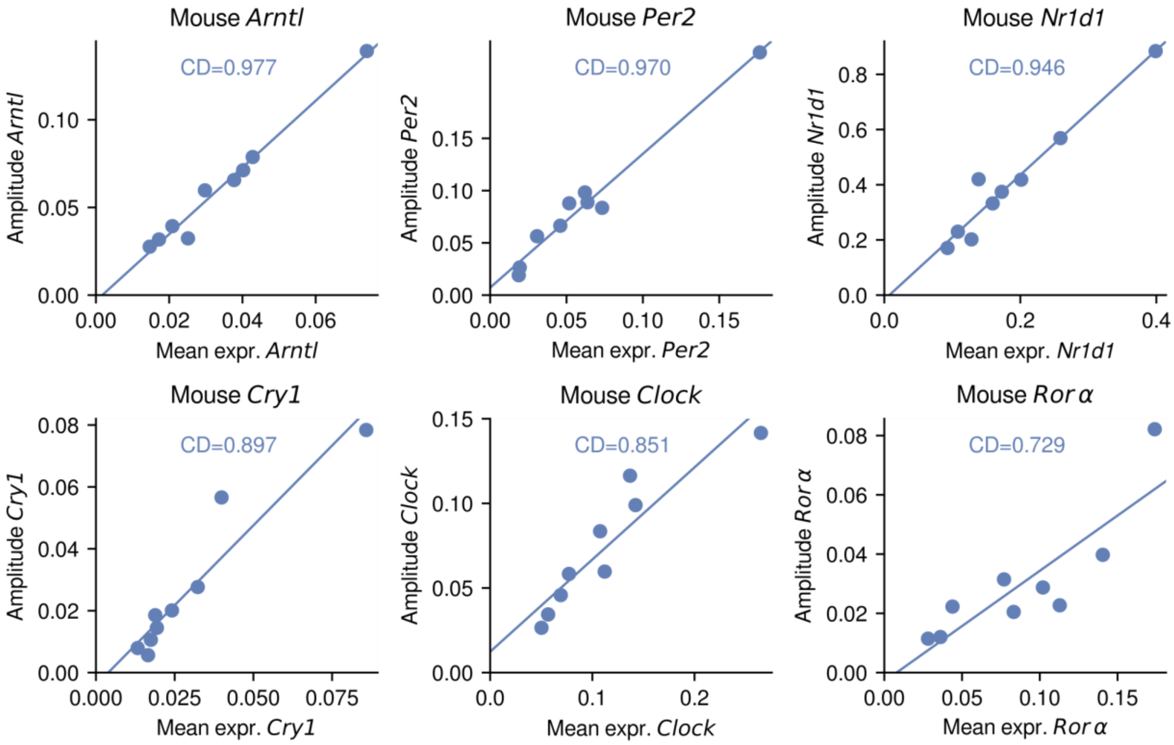

Supplementary Fig. 3: Correlation of amplitude and mean expression in different core-clock genes. Correlation (as measured by the coefficient of determination (CD) of the linear regression) is highest in the genes measured in this study, *Arntl, Per2* and *Nr1d1* (top row).

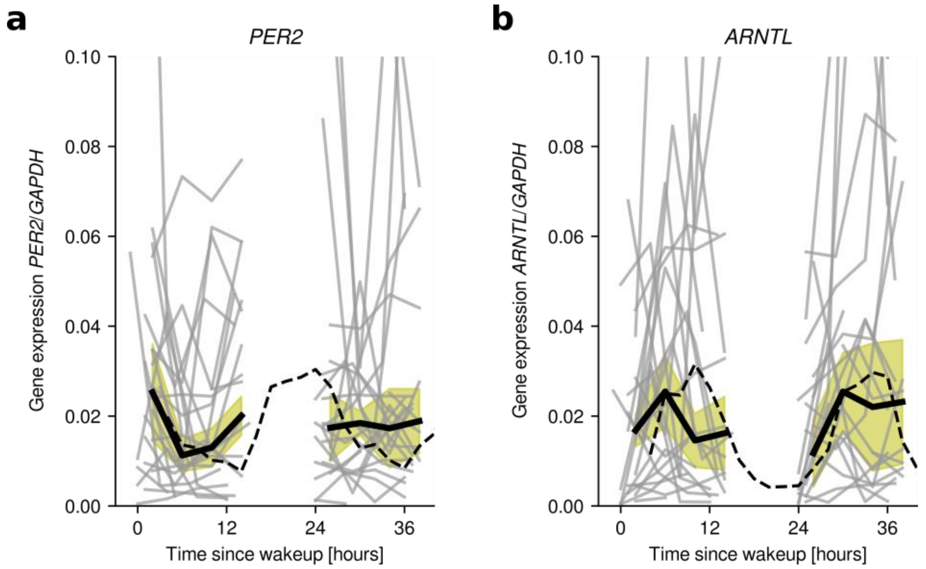

Supplementary Fig. 4: The absolute level of human gene expression fits to a mammalian reference. Gene expression of *PER2* and *Per2* **(a)** and *ARNTL1* and *Arntl* **(b)** relative to housekeeping gene *GAPDH and Gapdh* for human subjects and mouse, respectively. Time-series were aligned to the wake-up time of humans or lights off for mice (representing the start of nocturnal activity). Grey lines show the data for subjects measured on two consecutive days (excluding summer measurements). The mean over subject (black thick line, yellow area is plus/minus SEM) is similar to the circadian rhythm of the mouse heart, which is the mouse organ with the least oscillation amplitude.

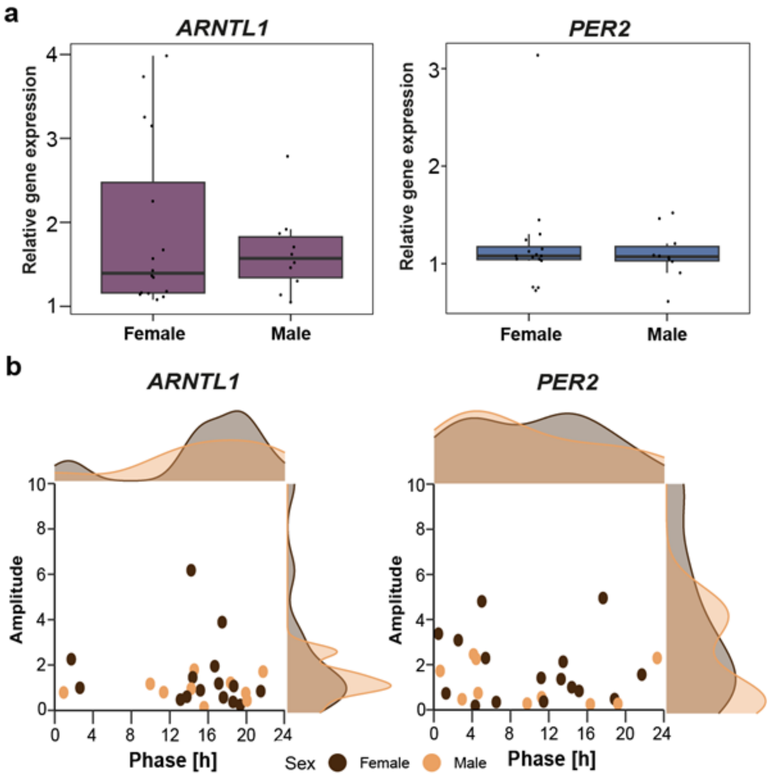

Supplementary Fig. 5: Sex-related differences in *ARNTL1* and *PER2* relative expression and circadian properties. a Box plot illustrating the relative expression levels of *ARNTL1* and *PER2* in male and female participants**. b** Acrophase and amplitude distribution of *ARNTL1* and *PER2* in male and female participants.

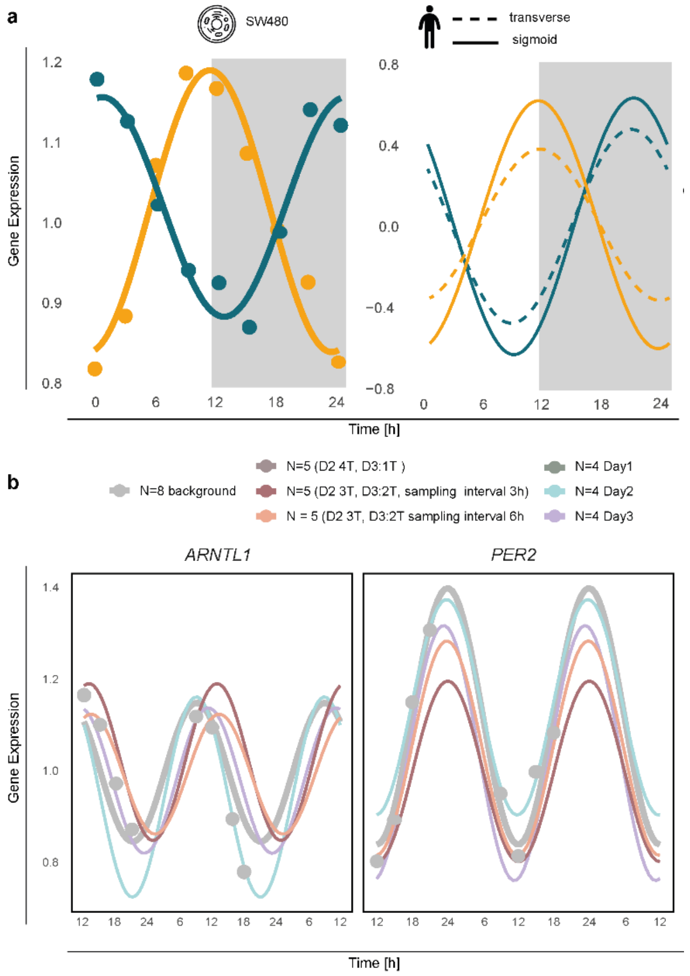

Supplementary Fig. 6: Circadian rhythms analysis using 24-h time course data. a. Depicted are the aligned harmonic regression (HR) fittings for *ARNTL (blue)* and *PER2* (orange) in SW480 CRC cells and Gtex human datasets (transverse and sigmoid colon tissues)[3,4]. **b.** Depicted harmonic regression curves for SW480 RNAseq dataset were generated with different number of data points (n=5; n=4) and time-intervals (start and end-points). n=8 corresponds to a background curve in grey.

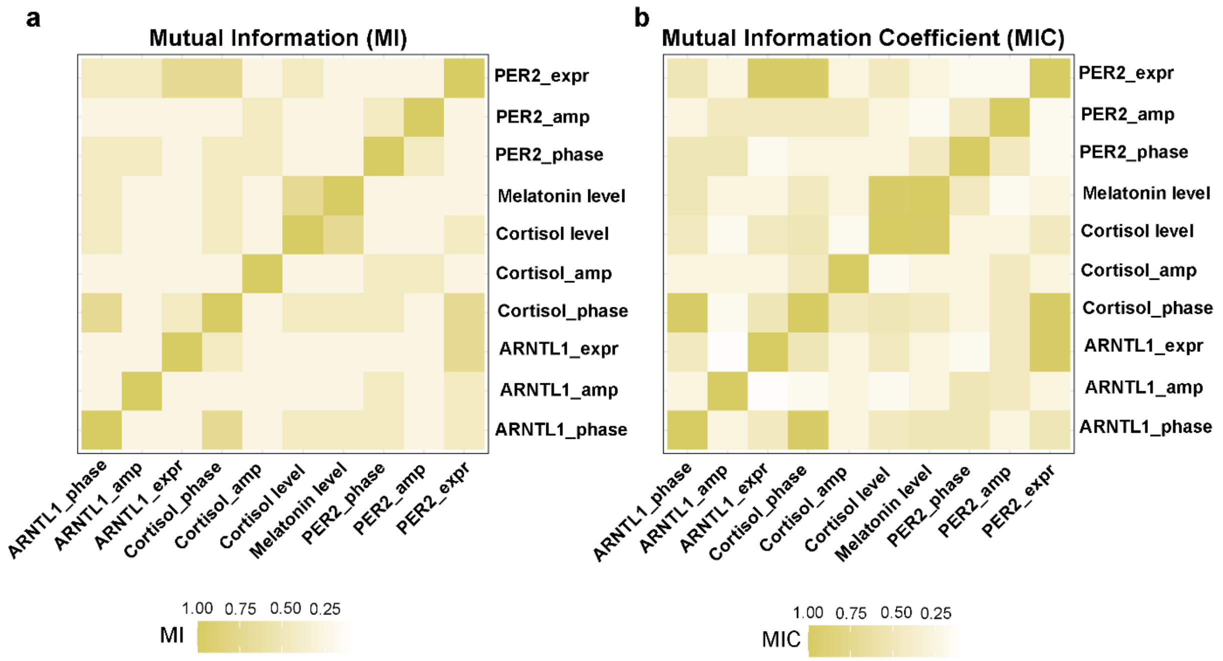

Supplementary Fig. 7: Heatmap Visualization of a Mutual Information (MI) and b Maximal Information Coefficient (MIC) Between Gene Expression and Hormonal Data. The heatmaps illustrate the relationships between various gene expression levels and hormonal data, with colour intensity representing the strength of the association.

References

1 Zhang R, L. N., Ballance HI, Hughes ME, Hogenesch JB. A circadian gene expression atlas in mammals: implications for biology and medicine. . *Proc Natl Acad Sci U S A.* **111**, 16219-16224, doi:doi: 10.1073/pnas.1408886111 (2014).

2 Mure LS, L. H., Benegiamo G, Chang MW, Rios L, Jillani N, Ngotho M, Kariuki T, Dkhissi-Benyahya O, Cooper HM, Panda S. Diurnal transcriptome atlas of a primate across major neural and peripheral tissues. . *Science* **359**, doi:doi: 10.1126/science.aao0318. (2018).

3 Talamanca, L., Gobet, C. & Naef, F. Sex-dimorphic and age-dependent organization of 24-hour gene expression rhythms in humans. *Science* **379**, 478-483, doi:10.1126/science.add0846 (2023).

4 El-Athman, R., Knezevic, D., Fuhr, L. & Relogio, A. A Computational Analysis of Alternative Splicing across Mammalian Tissues Reveals Circadian and Ultradian Rhythms in Splicing Events. *Int J Mol Sci* **20**, doi:10.3390/ijms20163977 (2019).
